# Chronic Hepatitis C in the Direct-Acting Antivirals Era: Carcinogenesis and Clinical Implications

**DOI:** 10.3390/diseases13120393

**Published:** 2025-12-05

**Authors:** Yucel Aydin, Ramazan Kurt, Veysel Tahan, Ebubekir Daglilar

**Affiliations:** 1 Section of Gastroenterology, Department of Internal Medicine, School of Medicine, Marmara University, Istanbul 34854, Türkiye; ramazan.kurt@marmara.edu.tr; 2 Division of Gastroenterology and Hepatology, Charleston Area Medical Center, West Virginia University, Charleston, WV 25304, USA; veysel.tahan@vandaliahealth.org (V.T.); ebubekir.daglilar@vandaliahealth.org (E.D.)

**Keywords:** hepatitis C virus, direct-acting antivirals, hepatocellular carcinoma, carcinogenesis, sustained virologic response, fibrosis, immune surveillance, viral reservoirs, prevention, elimination

## Abstract

Chronic hepatitis C virus (HCV) infection remains a major global health burden, responsible for substantial morbidity and mortality despite the advent of curative antiviral therapy. HCV induces hepatic injury and carcinogenesis through direct viral effects, persistent inflammation, oxidative stress, and metabolic disturbance. The introduction of direct-acting antivirals (DAAs) has revolutionized therapy, achieving sustained virologic response rates exceeding 95% and transforming HCV from a chronic, progressive disease into a curable infection. Nevertheless, viral eradication does not fully normalize hepatic or systemic risk. Patients with advanced fibrosis or cirrhosis continue to face an elevated incidence of hepatocellular carcinoma (HCC) and other complications, reinforcing the need for long-term monitoring. This review summarizes current knowledge of the molecular mechanisms underlying HCV-mediated carcinogenesis, the partial restoration of hepatic homeostasis following DAA-induced cure, and the clinical implications for surveillance and management in the post-HCV era. By integrating insights from molecular virology, immunopathogenesis, and clinical hepatology, the review highlights how persistent epigenetic and inflammatory footprints may sustain oncogenic potential even after viral clearance. A comprehensive understanding of these processes is essential for optimizing HCC prevention strategies, guiding surveillance policies, and advancing future therapeutic innovations aimed at complete hepatic recovery.

## 1. Introduction

Hepatitis C virus (HCV) represents one of the most significant infectious diseases of the modern era, with profound clinical, economic, and societal implications. More than three decades after its discovery in 1989 as the causative agent of what was historically termed non-A, non-B hepatitis, HCV continues to shape the field of hepatology and remains a catalyst for ongoing scientific inquiry [1,2]. The identification of HCV not only clarified the etiology of a previously enigmatic liver disease but also initiated in a new era of molecular virology, diagnostic innovation, and antiviral drug development. In recent years, advances in viral genomics and immunopathogenesis have further deepened our understanding of HCV biology, paving the way for novel therapeutic and preventive strategies beyond viral eradication [3,4]. Moreover, HCV has emerged as a paradigm for studying host–virus interactions, viral immune evasion, and mechanisms of chronicity, providing a uniquely informative model within the Flaviviridae family.

Globally, it is estimated that 58 million individuals are chronically infected with HCV, and approximately 1.5 million new infections occur annually [5]. Chronic infection is responsible for a substantial proportion of cirrhosis, decompensated liver disease, and hepatocellular carcinoma (HCC), and is implicated in more than 290,000 deaths each year [6]. The global burden of HCV remains uneven, with the highest prevalence in low- and middle-income countries where access to diagnosis and treatment remains limited, demonstrating the need for region-specific strategies. In addition, evolving epidemiologic patterns such as increased transmission through unsafe medical procedures in certain regions and rising incidence among young adults due to injection drug use, highlight the dynamic nature of the epidemic. In high-income nations, the opioid epidemic has substantially increased HCV transmission among people who inject drugs (PWID), contributing to a resurgence of new infections.

Importantly, the impact of HCV is not confined to hepatic morbidity and mortality. A growing body of evidence has firmly established the virus as a systemic pathogen that contributes to a diverse range of extrahepatic manifestations, including insulin resistance and type 2 diabetes, mixed cryoglobulinemia and B-cell lymphoproliferative disorders, chronic kidney disease, and neuropsychiatric complications [7,8]. These extrahepatic consequences significantly increase the overall disease burden and highlight the multisystemic nature of HCV infection. Recent studies suggest that extrahepatic manifestations may occur even in the early phases of infection and can persist despite viral clearance, reinforcing the notion that HCV induces widespread metabolic, immunologic, and vascular alterations.

The therapeutic journey of HCV is one of the most transformative narratives in modern medicine. For decades, treatment relied on interferon-based regimens, often in combination with ribavirin. These therapies were characterized by modest efficacy, prolonged duration, and considerable toxicity, which together limited widespread adoption and left most patients untreated. The introduction of direct-acting antivirals (DAAs) in the past decade revolutionized the field, achieving sustained virologic response (SVR) rates exceeding 95 percent across diverse patient populations [9,10,11]. This therapeutic revolution redefined HCV as a curable disease and raised, for the first time, the realistic prospect of global elimination. Beyond clinical cure, accumulating evidence suggests that DAAs can partially reverse hepatic fibrosis, improve metabolic homeostasis, and reduce extrahepatic inflammation though not all disease-related risks are fully normalized [12,13]. Notably, individuals with advanced fibrosis or cirrhosis continue to face residual risks even after achieving SVR, indicating persistent biological alterations that extend beyond viral clearance. These findings have stimulated intense research into epigenetic reprogramming, oncogenic pathway activation, and chronic immune dysregulation that may endure post-treatment and influence long-term outcomes.

Despite these unprecedented advances, several critical challenges remain. Most HCV-infected individuals worldwide remain undiagnosed, and even among those diagnosed, significant inequities in treatment access persist, particularly in low- and middle-income countries. Reinfection among high-risk groups, including PWID, continues to undermine elimination strategies. Furthermore, patients with advanced fibrosis or cirrhosis remain at persistent risk for HCC, even after achieving viral eradication, necessitating ongoing surveillance and long-term management. Emerging evidence suggests that epigenetic, metabolic, and immunologic alterations induced during chronic infection may persist after cure, potentially contributing to residual oncogenic risk in specific populations [14,15]. Furthermore, the absence of an effective prophylactic vaccine remains a major barrier to long-term control, pointing to the importance of sustained investment in vaccine research and prevention programs.

Recognizing both the progress achieved and the obstacles that remain, the World Health Organization (WHO) has announced elimination targets for 2030 with a 90 percent reduction in new HCV infections and a 65 percent reduction in HCV-related mortality [16]. Meeting these targets will require an integrated global effort encompassing prevention, screening, mitigation strategies, therapeutic expansion, and continued surveillance of long-term outcomes. It also demands sustained commitment to addressing healthcare inequities, expanding access to curative therapies, and implementing tailored interventions for vulnerable populations. As highlighted in the 2024 WHO Global Hepatitis Report, only a coordinated approach combining vaccination research, early detection, and equitable DAA distribution can sustain progress toward elimination [17]. In addition, innovative service delivery models such as decentralized testing, point-of-care diagnostics, and community-based treatment, are increasingly recognized as essential tools to accelerate progress, particularly in resource-limited settings.

Beyond its immediate impact, HCV serves as a paradigm for translational medicine. Its discovery, the rapid development of molecular diagnostics, and the unprecedented success of DAAs stand as landmarks in biomedical science. The trajectory of HCV research provides valuable lessons for other chronic viral infections, particularly hepatitis B virus (HBV) and human immunodeficiency virus (HIV), where the goals of cure or functional remission remain unmet. The experience with HCV reinforces the importance of integrating virology, drug discovery, global health policy, and patient-centered care that offers a blueprint for future therapeutic breakthroughs across infectious and non-infectious diseases alike. Moreover, HCV remains a valuable model for investigating how viral clearance interfaces with host repair pathways and cancer prevention, a topic of increasing significance in the DAA era [18,19]. These insights place HCV at the forefront of research into how chronic viral infections shape long-term immune and oncogenic pathways. These cross-disciplinary insights not only enhance our understanding of chronic viral pathogenesis but also expand the conceptual foundations of precision medicine and immuno-oncology.

This review provides a comprehensive synthesis of current knowledge on HCV, bridging insights from virology, molecular biology, clinical hepatology, oncology, and public health. We aim to highlight not only the scientific and therapeutic advances that have transformed HCV into a curable infection but also the enduring challenges that define the path toward elimination. By examining epidemiology, natural history, systemic manifestations, therapeutic strategies, and preventive measures, this article seeks to provide an integrated framework for understanding the ongoing and future impact of HCV on global health. Particular attention is given to the mechanisms of viral carcinogenesis, the incomplete restoration of hepatic homeostasis after cure, and the implications of these findings for HCC surveillance and prevention [20]. Through this integrated approach, the review aims to contextualize the evolving landscape of HCV research and its broader implications for hepatology and global health in the coming decades.

## 2. Global Burden and Epidemiology

### 2.1. Global Prevalence and Distribution

The epidemiology of HCV demonstrates substantial geographic heterogeneity, with important implications for targeted elimination efforts. Recent global estimates suggest a prevalence of approximately 0.8%, corresponding to nearly 58 million individuals living with chronic infection [21]. However, this burden is unevenly distributed, with distinct regions exhibiting substantially higher prevalence due to historical, healthcare-related, or behavioral risk factors.

Egypt remains a historical epicenter, with population-level prevalence exceeding 10% in some cohorts. This uniquely high burden is attributable to parenteral antischistosomal therapy campaigns conducted from the 1950s to the 1980s, during which extensive syringe reuse facilitated widespread iatrogenic transmission [22]. Similarly, Pakistan, Mongolia, and several Sub-Saharan African nations maintain high prevalence rates, driven by unsafe medical injections, inadequate infection control procedures, and limited access to sterilization technologies [23,24].

In contrast, North America, Western Europe, and Australia have witnessed a demographic shift toward younger individuals. In these regions, the opioid epidemic and escalating injection drug use have become the dominant drivers of new infections, with PWID representing the highest-risk population [25,26]. Reinfection among PWID following successful therapy remains a substantial barrier to elimination and necessitates robust harm-reduction strategies.

In the United States between 2017 and 2020, an estimated 2.2 million adults (0.9%) were living with active HCV infection defined by detectable RNA, though adjusted models accounting for underrepresented PWID populations place the prevalence closer to 1.6%. In the European Union/European Economic Area, surveillance data from 2022 documented 23,249 new diagnoses across 29 countries (excluding those reporting acute infections only), corresponding to an incidence of 6.2 cases per 100,000 population. A summary of key epidemiological indicators across major global regions is presented in Table 1 to provide a comparative overview of prevalence, incidence, and dominant transmission patterns.

### 2.2. Transmission Dynamics

HCV is transmitted primarily through direct percutaneous exposure to infected blood. Historically, transmission was most strongly associated with unsafe transfusion practices, inadequate sterilization of medical equipment, and iatrogenic exposures. With the widespread adoption of universal blood screening, nucleic acid testing, and stringent infection-control protocols in high-income settings, transfusion-related transmission has been virtually eliminated [27].

In the contemporary era, injection drug use is the predominant mode of transmission, accounting for more than 60 percent of new infections in many regions [28]. Additional risk factors include unsterile tattooing and body piercing practices, reuse or improper disinfection of hemodialysis equipment, and occupational exposures among healthcare workers. While sexual transmission occurs less frequently, it is increasingly recognized in populations with high-risk sexual practices, particularly among men who have sex with men. Vertical transmission from mother to child occurs in approximately 5 to 7 percent of pregnancies, with the risk significantly elevated among women co-infected with HIV [29].

Understanding these evolving patterns of transmission is essential for shaping effective public health strategies, including risk-reduction interventions, infection-control measures, and targeted screening programs. Such efforts are central to the global elimination agenda and provide the foundation for interpreting regional variation in HCV epidemiology.

### 2.3. Progress Toward Elimination

The advent of DAAs, which consistently deliver cure rates above 95% across diverse patient populations, has transformed the global pursuit of hepatitis C elimination [9,10,11]. Building on this progress, the WHO established bold 2030 elimination objectives in 2016, seeking a 90% reduction in new chronic infections and a 65% decrease in deaths attributable to HCV [5,16].

According to the 2024 WHO Global Hepatitis Report, most countries remain off-track to meet these targets. Globally, approximately 30% of infected individuals are diagnosed, and fewer than 20% have received treatment, highlighting major gaps in screening and care access [5]. Low- and middle-income countries face the most substantial challenges, including high diagnostic costs, limited laboratory capacity, workforce shortages, and fragmented care systems [21,23]. Even in high-income nations, under-diagnosis among marginalized groups such as PWID, incarcerated individuals, and the unstably housed, continues to impede progress [25,26].

Despite these challenges, several countries have demonstrated measurable success. Egypt, Georgia, Iceland, and Australia have implemented national elimination strategies combining universal DAA access, community-based screening programs, micro-elimination approaches, and integrated risk-mitigation services [22,24]. These models have produced substantial declines in incidence, fibrosis progression, and liver-related mortality.

Mathematical modeling indicates that achieving elimination requires annual treatment rates of at least 80 per 100,000 population, expanded risk-mitigation services, and widespread adoption of point-of-care diagnostics [7,21]. Reinfection among PWID remains a substantial barrier but can be mitigated through opioid agonist therapy, needle/syringe exchange programs, and peer-led community outreach [25,28].

While global elimination is an achievable goal, it will require sustained political commitment, equitable treatment access, targeted intervention for vulnerable populations, and ongoing surveillance to monitor long-term outcomes [5,16].

## 3. Virology and Molecular Biology

### 3.1. Viral Genome and Structure

HCV is a positive-sense, single-stranded RNA virus of approximately 9.6 kilobases, classified within the genus Hepacivirus of the Flaviviridae family [30]. The viral genome contains a single open reading frame (ORF) flanked by highly structured untranslated regions (UTRs) at both termini. The 5′ UTR harbors a type III internal ribosome entry site (IRES) that mediates cap-independent translation, enabling efficient viral protein synthesis even when host cell translation is suppressed [31]. The 3′ UTR contains variable, poly(U/UC), and X-tail stem-loop domains, all of which regulate RNA stability and replication complex assembly [31].

Translation of the viral genome produces a 3000-amino-acid polyprotein that undergoes co- and post-translational cleavage by host signal peptidases and viral proteases to generate ten mature viral proteins [30]. These include three structural proteins—core, E1, and E2; the p7 viroporin; and six non-structural proteins (NS2, NS3, NS4A, NS4B, NS5A, NS5B). Core protein, a highly basic RNA-binding protein, forms the nucleocapsid and interacts with lipid droplets, contributing to hepatic steatosis and metabolic dysregulation [32]. The E1 and E2 envelope glycoproteins assemble into noncovalent heterodimers that mediate receptor binding and membrane fusion; E2 contains hypervariable regions, especially HVR1, which enable rapid escape from neutralizing antibodies [33].

The p7 protein, functioning as a viroporin, regulates ion conductance essential for efficient assembly and secretion of infectious virions [30]. NS2 acts as a cysteine protease that initiates virion assembly by coordinating interactions between structural and nonstructural proteins. NS3, a multifunctional serine protease and helicase, plays a central role in polyprotein processing and RNA unwinding, with activity enhanced by its membrane-anchoring cofactor NS4A [30].

NS4B remodels endoplasmic reticulum membranes to generate the membranous web, a defining feature of the viral replication complex. NS5A, a pleiotropic phosphoprotein, is involved in RNA replication, particle assembly, and modulation of host antiviral signaling pathways [31]. NS5B, the viral RNA-dependent RNA polymerase, catalyzes RNA synthesis and is the major target of nucleotide analog inhibitors.

**Figure 1:**
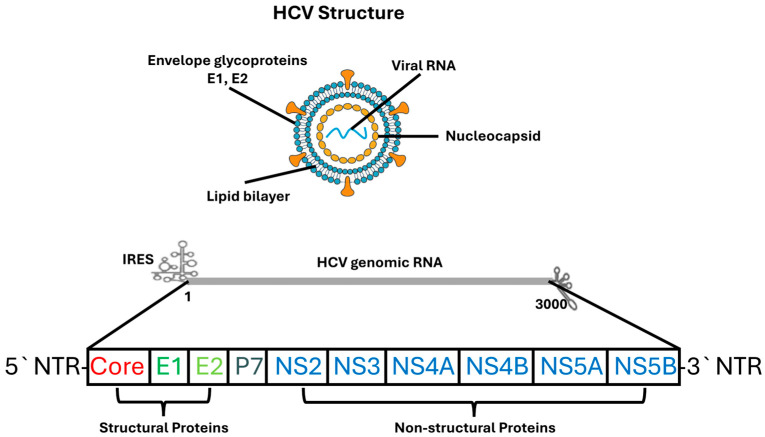
Structure and Genomic Organization of the HCV. The upper panel illustrates the architecture of the HCV virion, which consists of a lipid bilayer envelope embedded with the viral glycoproteins E1 and E2, surrounding an internal nucleocapsid composed of core protein and positive-sense single-stranded RNA. The lower panel depicts the organization of the HCV genome, including the 5′ and 3′ NTRs, IRES, and the single open reading frame encoding structural proteins (Core, E1, E2, p7) and non-structural proteins (NS2, NS3, NS4A, NS4B, NS5A, NS5B). Structural proteins are involved in virion formation and entry, whereas non-structural proteins participate in viral replication, assembly, and host–cell modulation.

Recent advances in structural virology, particularly high-resolution cryo-electron microscopy, have revealed detailed conformations of NS5A, insights into NS5B polymerase dynamics during RNA synthesis, and the architecture of the E1/E2 heterodimer [31]. These findings have substantially deepened understanding of HCV replication biology and facilitated rational development of direct-acting antivirals. Figure 1 provides a schematic representation of the HCV particle structure and its genomic organization, illustrating the major structural and non-structural proteins discussed in this section.

### 3.2. Genetic Diversity

HCV displays exceptional genetic diversity, making it one of the most heterogeneous human RNA viruses. This diversity is driven by the virus’s extraordinarily high replication rate with 10^12^ virions produced each day along with the low-fidelity nature of the NS5B RNA-dependent RNA polymerase, which lacks proofreading activity [32]. As a result, infected individuals harbor a dynamic mixture of closely related viral variants, known as quasispecies, that continually evolve under selective pressures imposed by host immunity and antiviral therapy. This inherent variability facilitates immune evasion, persistence, and adaptation to different ecological and epidemiological settings.

Phylogenetic studies classify HCV into seven major genotypes and more than sixty subtypes, which differ significantly at the nucleotide level and exhibit distinct global distributions [32]. Genotype 1 is the most widespread globally and is predominant in North America, South America, and much of Western Europe. Genotype 3 is especially common in South Asia, where historical medical practices involving unsterile injection equipment contributed to its extensive spread. Genotype 4 is concentrated in the Middle East and Sub-Saharan Africa, while genotypes 5 and 6 have more localized distributions, primarily in South Africa and Southeast Asia, respectively. Genotype 7, which has been more recently identified, has been documented in Central Africa. These patterns reflect historical transmission routes, demographic shifts, and region-specific healthcare practices. The global distribution and key characteristics of HCV genotypes are summarized in Table 2.

A key contributor to HCV’s genetic plasticity is the envelope glycoprotein E2, particularly its hypervariable region 1 (HVR1). This region undergoes rapid immune-driven evolution, allowing the virus to evade neutralizing antibodies and shaping much of the observed sequence diversity [33]. The antigenic variability of HVR1 presents a formidable challenge to vaccine development, as conserved epitopes are often obscured by the surrounding variable residues, limiting the durability of humoral immune responses.

Before the advent of DAAs, viral genotypes had profound clinical implications. Interferon-based therapies were strongly genotype-dependent, with significantly lower SVR rates in individuals infected with genotypes 1 and 4 compared with genotypes 2 and 3. Although modern DAA regimens have dramatically narrowed these disparities, genotype 3 continues to pose unique therapeutic challenges, including higher rates of hepatic steatosis, accelerated fibrosis progression, and slightly reduced SVR in patients with advanced liver disease. These observations suggest that the biological characteristics of genotype 3 may influence pathogenesis in ways distinct from other genotypes.

Advances in next-generation sequencing have further refined understanding of intra-host viral evolution, revealing selective pressures that shape the emergence of resistance-associated substitutions (RASs) in viral nonstructural proteins targeted by DAAs. While the high efficacy of pan-genotypic DAA regimens has mitigated the clinical impact of many RASs, ongoing surveillance remains important, particularly in regions where specific subtypes predominate.

Recent updates from the International Committee on Taxonomy of Viruses have expanded the classification of *Flaviviridae*, incorporating newly discovered hepaciviruses in non-human species and providing deeper insight into the evolutionary origins of HCV diversity [33]. These developments continue to inform molecular epidemiology, improve understanding of viral evolution, and support ongoing efforts in vaccine research.

### 3.3. Replication Cycle

The replication cycle of HCV is intricately linked to hepatocyte membrane dynamics, host lipid homeostasis, and intracellular organelle remodeling. Infection begins when circulating HCV particles interact with hepatocyte surface molecules. Initial attachment occurs through low-density lipoprotein receptor (LDLR) and scavenger receptor class B type I (SR-B1), which facilitate concentration of viral particles at the cell surface. This is followed by a highly coordinated sequence of receptor engagements involving CD81, claudin-1, and occludin. Recent imaging and structural studies have clarified the central role of CD81–claudin-1 complexes in orchestrating membrane fusion, revealing dynamic conformational transitions required for viral internalization [34]. These receptor interactions guide the virus into the clathrin-mediated endocytic pathway, where acidification of the endosome triggers fusion of the viral envelope with the endosomal membrane and release of the positive-sense RNA genome into the cytoplasm.

Once uncoated, the viral RNA is immediately translated at endoplasmic reticulum (ER)–associated ribosomes, producing the viral polyprotein. Co- and post-translational cleavage by host signal peptidases and viral proteases generates the mature structural and nonstructural proteins. Viral RNA replication occurs on specialized ER-derived structures known collectively as the membranous web, which is induced primarily by NS4B. This dynamic network of single- and double-membrane vesicles provides a physically protected environment that shelters viral RNA from cytosolic innate immune sensors and supports efficient replication [35].

The assembly of the replicase complex involves NS3/4A, NS5A, and NS5B, along with multiple host proteins. Cyclophilin A, PI4KIIIα, and SEC14L2 contribute to the formation and stabilization of replication organelles, while microRNA-122, the most abundant liver-specific microRNA, binds to the 5′ UTR of the viral genome and enhances RNA stability and replication efficiency [35,36]. NS5B synthesizes a complementary negative-strand RNA, which serves as the template for producing new positive-strand genomes. These replication intermediates remain tightly associated with the membranous web to maintain replication fidelity and prevent recognition by RIG-I and MDA5.

**Figure 2:**
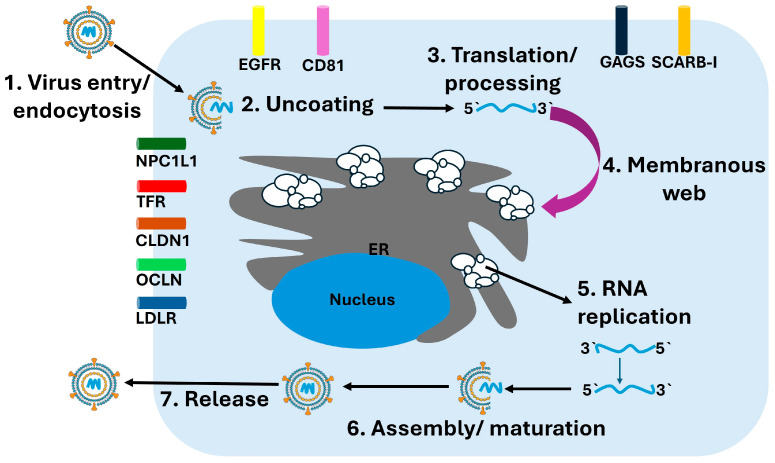
HCV Replication Cycle. The schematic illustrates the major stages of the HCV replication cycle within hepatocytes. (1) Virus entry occurs through receptor-mediated endocytosis involving multiple host factors including CD81, CLDN1, OCLN, LDLR, NPC1L1, TFR, and EGFR. (2) Following endosomal fusion, the viral particle undergoes uncoating, releasing the positive-sense RNA genome. (3) The viral RNA is translated at the rough endoplasmic reticulum (ER) into a single polyprotein, which undergoes proteolytic processing into structural and nonstructural proteins. (4) Viral nonstructural proteins remodel the ER membrane to form the membranous web, the platform for replication. (5) Within this structure, RNA replication occurs via synthesis of a negative-strand intermediate that serves as a template for new positive-strand genomes. (6) Newly synthesized genomes are packaged with core protein and envelope glycoproteins during assembly and maturation. (7) Mature virions are released through the secretory pathway.

Assembly of new viral particles occurs near ER–lipid droplet junctions, where the core protein associates with lipid droplets and recruits nascent viral genomes along with nonstructural proteins needed for packaging. This process is intimately linked to hepatic lipid metabolism. Apolipoproteins, especially apolipoprotein E (ApoE), are incorporated into assembling virions and are essential for maturation, infectivity, and hepatotropism. Mature virions exit the cell via the very-low-density lipoprotein (VLDL) secretion pathway, generating hybrid lipoviroparticles that facilitate immune evasion and efficient dissemination within the host [37]. An overview of the hepatitis C virus replication cycle is depicted in Figure 2, illustrating the sequential steps of viral entry, translation, RNA replication, assembly, and release.

The interplay between viral replication and host lipid metabolism contributes to several pathological features of chronic HCV infection, including hepatic steatosis, ER stress, oxidative injury, and inflammatory signaling. These processes create a microenvironment that promotes fibrogenesis and carcinogenesis. Additionally, the remodeling of intracellular membranes during replication alters autophagy, mitochondrial dynamics, and lipid trafficking, further shaping the pathogenic landscape associated with persistent infection [36,37].

### 3.4. Immune Evasion

HCV establishes persistent infection in the majority of exposed individuals through an intricate array of mechanisms that disable innate antiviral defenses and progressively erode adaptive immunity. One of the earliest immune-evasion strategies involves the structural composition of the viral envelope glycoproteins. The E2 glycoprotein contains HVR1 which exhibit rapid sequence diversification driven by antibody-mediated selection. This continuous remodeling of antigenic epitopes prevents effective neutralization and allows circulating quasispecies to escape antibody pressure, thereby undermining durable humoral immunity [38]. Additionally, the density and glycosylation of E1/E2 complexes on the virion surface obscure conserved epitopes, limiting the ability of broadly neutralizing antibodies to recognize and neutralize the virus.

A hallmark of HCV immune evasion is its potent inhibition of innate immune signaling. The NS3/4A protease cleaves two key adaptor proteins; MAVS, located on the mitochondrial outer membrane, and TRIF, a central component of TLR3 pathways. Cleavage of these molecules prevents downstream activation of IRF3 and NF-κB, thereby suppressing the production of type I interferons and interferon-stimulated genes (ISGs) [38]. This disruption of early antiviral responses enables the virus to replicate within hepatocytes with minimal initial immune recognition. NS5A also interferes with innate signaling by binding and inhibiting PKR, modulating STAT1 phosphorylation, and disrupting cellular stress-response pathways that normally restrict viral replication [39].

As infection becomes chronic, profound changes in adaptive immunity emerge. CD8^+^ T cells, initially responsible for controlling viral replication, progressively develop a state of functional exhaustion characterized by diminished cytokine secretion, reduced proliferative capacity, and high expression of inhibitory receptors such as PD-1, TIM-3, and LAG-3 [39]. Intrahepatic CD8^+^ T cells often display an exhaustion transcriptional signature that persists even after viral clearance, suggesting durable epigenetic reprogramming resulting from prolonged antigen exposure [40]. This exhausted phenotype is paralleled by impaired CD4^+^ T helper cell support, further weakening antiviral effector function.

In addition to T-cell exhaustion, HCV manipulates dendritic cell (DC) function by reducing their ability to prime naive T cells and by altering cytokine secretion profiles. Natural killer (NK) cell activity is also modulated, with chronic infection associated with phenotypes showing decreased cytotoxicity and altered expression of activating receptors. Moreover, expansion of regulatory T cells promotes an immunosuppressive hepatic environment, limiting antiviral immune responses while simultaneously contributing to chronic inflammation and fibrogenesis.

Together, these evasion strategies enable the virus to persist despite robust host immune defenses. They also shape the immunopathogenesis of chronic infection by promoting long-term inflammation, fibrotic remodeling, and an immunologically permissive microenvironment that contributes to hepatocarcinogenesis [41]. Even after antiviral cure, remnants of these immune alterations persist, helping to explain the ongoing risk of hepatocellular carcinoma in individuals with advanced fibrosis or cirrhosis.

## 4. Natural History of HCV Infection

### 4.1. Acute Infection and Spontaneous Clearance

Acute HCV infection is frequently clinically silent, with most individuals remaining asymptomatic during the early phase. Only about 20 to 30 percent of patients develop jaundice or clinically apparent hepatitis, which often leads to delayed diagnosis [42]. When symptoms occur, they are typically nonspecific, including such symptoms as fatigue, malaise, or mild right upper quadrant discomfort, and are rarely severe enough to prompt early medical evaluation.

Spontaneous viral clearance occurs in approximately 15 to 25 percent of infected individuals, usually within the first six months of infection. Successful clearance is associated with a vigorous and sustained host immune response, including such mechanisms as activation of CD4^+^ helper T cells, CD8^+^ cytotoxic T cells, and robust type I interferon signaling [43]. In individuals who do not mount such responses, viremia persists and chronic infection is established.

Host genetic polymorphisms exert a strong influence on the likelihood of clearance. Variants in the IL28B (IFNL3) gene locus, which encodes interferon lambda 3, have been shown to modulate both spontaneous and treatment-induced viral clearance, emphasizing the importance of innate immune pathways in shaping clinical outcomes [44].

In the absence of spontaneous clearance, approximately 75 to 85 percent of patients develop chronic HCV infection, which represents the basis of the global disease burden and drives the risk of progressive liver injury, cirrhosis, and HCC.

### 4.2. Chronic Infection and Fibrosis Progression

Most individuals, approximately 75 to 85 percent, progress from acute to chronic HCV infection, which is characterized by persistent hepatocellular injury, ongoing necroinflammation, and activation of fibrogenic pathways [45]. Over time, these processes promote the deposition of extracellular matrix proteins, leading to progressive fibrosis and architectural distortion of the liver.

The rate of fibrosis progression is highly variable and influenced by both viral and host-related factors. Key determinants include age at the time of infection, alcohol consumption, co-infection with other viruses such as HIV or HBV, and metabolic conditions including obesity and insulin resistance. Male sex has also been consistently associated with faster progression [46].

In many patients, advanced fibrosis and cirrhosis develop within 20 to 30 years of persistent infection. However, a subset of individuals, often referred to as rapid progressors, may progress to cirrhosis in less than a decade, particularly in the presence of compounding risk factors such as heavy alcohol use, HIV co-infection, or advanced age at acquisition. The identification of such high-risk groups remains essential for guiding early intervention and surveillance strategies.

### 4.3. Cirrhosis, Decompensation, and HCC

Once cirrhosis is established, the risk of clinical decompensation becomes a defining feature of disease progression. The annual incidence of decompensation ranges between 2 and 5 percent, with complications including ascites, variceal hemorrhage, hepatic encephalopathy, and jaundice [47]. These events mark the transition from compensated to decompensated cirrhosis and are associated with markedly reduced survival, stressing the importance of early recognition and management.

In addition to decompensation, cirrhosis confers a substantial risk of HCC. Among patients with established cirrhosis, the annual incidence of HCC is estimated at 2 to 4 percent [48]. The pathogenesis of HCC in this setting reflects a complex interplay of chronic necroinflammation, fibrosis, and direct viral effects on hepatocarcinogenesis. Importantly, the advent of DAAs has transformed outcomes by achieving SVR in the vast majority of patients. However, even after viral eradication, individuals with advanced fibrosis or cirrhosis retain a residual lifelong risk of HCC, necessitating continued surveillance with imaging and biomarkers. This residual risk highlights the need for ongoing hepatocellular carcinoma screening programs, even in the era of curative therapy.

Beyond these hepatic outcomes, HCV also exerts a profound impact on multiple extrahepatic organ systems, contributing to metabolic, immunological, renal, and neurological complications that further expand the clinical and societal burden of the infection [49].

## 5. Extrahepatic Manifestations of HCV Infection

Although HCV is primarily a hepatotropic virus, it is increasingly recognized as a systemic pathogen with diverse and clinically significant effects beyond the liver. Epidemiological studies indicate that 40 to 70 percent of patients develop at least one extrahepatic manifestation during infection [50]. These conditions arise from chronic immune activation, deposition of circulating immune complexes, and metabolic derangements, and they collectively contribute to the substantial morbidity associated with chronic infection.

### 5.1. Mixed Cryoglobulinemia and Vasculitis

Mixed cryoglobulinemia (MC) represents one of the most clinically significant extrahepatic manifestations of chronic HCV infection. It is characterized by circulating immune complexes composed of polyclonal IgG and monoclonal or polyclonal IgM with rheumatoid factor activity that precipitate at low temperatures and dissolve on warming. These immune complexes deposit in small- and medium-sized vessels, leading to systemic vasculitis that predominantly affects the skin, kidneys, and peripheral nerves. The classical histopathologic finding is leukocytoclastic vasculitis with complement activation and endothelial injury.

HCV-driven B-cell stimulation and the persistent presence of viral antigens play key roles in disease pathogenesis. Chronic antigenic stimulation promotes expansion of B-cell clones producing cryoglobulins, a process often associated with elevated serum rheumatoid factor and hypocomplementemia [51,52]. Next-generation sequencing studies have demonstrated that these B-cell expansions frequently exhibit stereotyped use of immunoglobulin variable-region genes, supporting the concept that MC reflects an antigen-driven, oligoclonal B-cell disorder with malignant potential [53].

Clinically, patients present with palpable purpura, arthralgia, fatigue, and peripheral neuropathy, forming the classic Meltzer triad. Renal involvement, most commonly type I membranoproliferative glomerulonephritis, remains the main determinant of prognosis. Although DAA therapy achieves viral eradication in nearly all patients, cryoglobulinemic vasculitis may persist or relapse in a minority. Persistence is frequently linked to residual B-cell clonal expansions and incomplete immunologic normalization despite virologic cure [54,55,56]. These findings highlight the importance of long-term monitoring and, in refractory cases, the use of B-cell-directed therapies such as rituximab.

### 5.2. Lymphoproliferative Disorders

Chronic HCV infection is strongly associated with B-cell lymphoproliferative disorders, ranging from benign monoclonal expansions to overt non-Hodgkin lymphoma (NHL). The pathogenetic link is rooted in persistent antigenic stimulation by viral proteins and immune complexes that promote continuous B-cell activation, somatic hypermutation, and clonal selection. A continuum exists between mixed cryoglobulinemia and lymphoma, as B-cell clones producing rheumatoid factor-like IgM may evolve toward monoclonality over time [57,58].

Epidemiologic data consistently demonstrate a two- to three-fold increased risk of B-cell NHL in HCV-infected individuals, particularly marginal zone lymphoma, lymphoplasmacytic lymphoma, and diffuse large B-cell lymphoma [59]. Molecular studies have shown stereotyped immunoglobulin heavy-chain rearrangements, constitutive NF-κB pathway activation, and epigenetic alterations, providing mechanistic support for chronic antigen-driven lymphomagenesis [60,61].

Eradication of HCV with DAAs leads to hematologic remission in many patients with indolent lymphomas, reinforcing the causal relationship between viral persistence and lymphoproliferation. However, some cases persist or relapse after successful viral clearance, suggesting that autonomous B-cell clones and long-standing epigenetic reprogramming can maintain lymphomagenic activity independent of antigenic stimulation [62]. These findings highlight the importance of ongoing hematologic surveillance even after achieving sustained virologic response.

### 5.3. Metabolic and Endocrine Disorders

Chronic HCV infection has been implicated in a spectrum of metabolic complications, including insulin resistance, type 2 diabetes mellitus, and hepatic steatosis. These effects are mediated through interference with insulin signaling pathways and alterations in host lipid metabolism [63]. Notably, genotype 3 is strongly associated with steatosis, reflecting its direct impact on lipid droplet biology and VLDL secretion. These metabolic sequelae not only accelerate fibrosis progression but also contribute to increased cardiovascular morbidity among patients with chronic HCV [64].

### 5.4. Neuropsychiatric Manifestations

Patients with chronic HCV infection frequently experience neuropsychiatric symptoms, including fatigue, depression, cognitive impairment, and sleep disturbances [65]. While interferon-based therapy historically exacerbated psychiatric morbidity, accumulating evidence indicates that HCV itself contributes to neuroinflammation, alteration of serotonin metabolism, and activation of microglia within the central nervous system [66]. These findings emphasize the need for integrated neuropsychiatric assessment and care as a fundamental component of comprehensive HCV management.

## 6. HCV and Hepatocarcinogenesis

### 6.1. Mechanisms of HCV-Induced HCC

HCV-associated HCC arises from a complex interplay of direct viral effects and indirect consequences of chronic inflammation and fibrosis. In contrast to HBV, HCV does not integrate into the host genome. Instead, hepatocarcinogenesis is mediated by a combination of viral protein activity, persistent immune-mediated injury, and microenvironmental alterations that together establish a carcinogenic milieu.

Among viral proteins, the HCV core protein exerts potent oncogenic activity. It modulates apoptotic pathways, cell cycle regulation, and lipid metabolism, promoting steatosis, oxidative stress, and genomic instability [67]. Similarly, NS5A interacts with host signaling pathways, including p53, retinoblastoma protein, and Wnt/β-catenin, and influences angiogenesis, thereby facilitating malignant transformation [68].

The indirect oncogenic effects of chronic HCV infection are equally important. Persistent necroinflammation drives hepatocyte death and regeneration, increasing the risk of DNA damage and accumulation of oncogenic mutations. Progressive fibrosis and cirrhosis provide a pro-carcinogenic microenvironment by altering extracellular matrix architecture and promoting cellular dysplasia [69].

Additional mechanisms contribute to HCV-related hepatocarcinogenesis, including epigenetic modifications, mitochondrial dysfunction, and dysregulation of microRNAs, most notably the liver-specific microRNA miR-122, which plays a critical role in both viral replication and hepatocyte transformation [70]. Taken together, these direct and indirect pathways explain the persistent risk of HCC in patients with advanced fibrosis or cirrhosis, even after viral eradication with direct-acting antivirals.

### 6.2. HCC Risk After DAA Therapy

The achievement of SVR with DAAs represents a major therapeutic milestone and is associated with a substantial reduction in the risk of HCC. However, SVR does not abolish oncogenic potential. A persistent residual risk remains in patients with advanced fibrosis or cirrhosis, reflecting the cumulative field effect of chronic liver injury, the irreversible architectural changes in cirrhosis, and lingering molecular alterations induced by long-standing infection in addition to persistent post-viral signature on the genes of hepatocytes [71].

Early observational studies raised concern about a paradoxical increase in de novo HCC incidence following DAA therapy, leading to debate over whether viral eradication altered tumor surveillance or immune responses in a manner that promoted carcinogenesis. However, subsequent large-scale cohort studies and meta-analyses have confirmed that DAAs significantly reduce both the incidence of HCC and the recurrence of tumors after curative therapy, findings that are consistent with the virological and clinical benefits of viral eradication [72].

Despite these gains, the residual lifelong risk of HCC necessitates continued surveillance in all patients with advanced fibrosis or cirrhosis, even after viral cure. Standard practice remains semiannual imaging, with or without serum biomarkers, to ensure early detection and timely management of HCC in this high-risk population Despite these gains, lifelong surveillance remains essential in all patients with advanced fibrosis or cirrhosis, even after viral cure. Semiannual imaging, with or without serum biomarkers, remains the standard for early HCC detection. Recent risk-stratification models incorporating fibrosis stage, metabolic traits, and genetic/epigenetic signatures may further improve surveillance precision in the post-SVR population [73,74].

## 7. Advances in Antiviral Therapy

### 7.1. Evolution of Treatment

The therapeutic landscape of HCV has undergone remarkable transformation over the past three decades. Early treatment efforts relied on interferon monotherapy, which achieved SVR rates of only 10 to 20 percent and was associated with substantial toxicity, including flu-like symptoms, cytopenia, and neuropsychiatric complications [75]. The subsequent addition of ribavirin provided a modest improvement in efficacy, but treatment remained poorly tolerated and was feasible for only a subset of motivated patients.

The introduction of pegylated interferon in combination with ribavirin represented the next major advance, prolonging interferon’s half-life and enhancing treatment potency. Nevertheless, SVR rates remained suboptimal, and adverse effects continued to limit widespread uptake. The therapeutic paradigm shifted further with the arrival of the first-generation protease inhibitors boceprevir and telaprevir, which were used in combination with pegylated interferon and ribavirin. These triple therapy regimens significantly improved SVR rates, particularly in genotype 1 infection, but were hampered by complex dosing schedules, frequent adverse events, and the rapid emergence of drug resistance [76].

### 7.2. Direct-Acting Antivirals

The advent of DAAs revolutionized the management of HCV, transforming it from a chronic, often progressive infection into a curable disease. These agents are administered orally, are well tolerated, and achieve SVR rates exceeding 95 percent across all major genotypes, representing a dramatic improvement over interferon-based regimens. DAAs act by targeting nonstructural viral proteins that are essential for replication, thereby interrupting key steps in the viral life cycle [77].

Three principal classes of DAAs form the backbone of modern therapy. The NS3/4A protease inhibitors, such as grazoprevir, glecaprevir, and voxilaprevir, block polyprotein processing and prevent viral maturation. The NS5A inhibitors, including ledipasvir, velpatasvir, and pibrentasvir, interfere with both RNA replication and virion assembly. The NS5B polymerase inhibitors, such as sofosbuvir (a nucleotide analogue) and dasabuvir (a non-nucleoside inhibitor), directly inhibit the viral RNA-dependent RNA polymerase and halt genome replication.

The development of fixed-dose combination regimens has further simplified therapy and expanded global access. Widely used examples include sofosbuvir/velpatasvir and glecaprevir/pibrentasvir, both of which provide pangenotypic coverage. These regimens are administered as short courses of eight to twelve weeks, with minimal pill burden and excellent tolerability, enabling treatment scale-up in diverse healthcare settings.

Unlike interferon-based regimens, DAA therapy is interferon-free, highly tolerable, and does not require response-guided adjustments. Treatment can now be delivered in standardized short courses without the hematologic, psychiatric, and systemic toxicities that historically limited patient uptake. This transformation has not only redefined the standard of care for HCV but has also provided a model for the rapid development of curative therapies in other chronic viral infections [78].

### 7.3. Special Populations

The safety and efficacy of DAAs extend to populations that were historically considered difficult to treat. Clinical trials and real-world studies have consistently demonstrated that DAAs achieve high rates of sustained virologic response in individuals with HIV co-infection, patients with decompensated cirrhosis, those with renal impairment including individuals on dialysis, and recipients of liver or kidney transplantation [79]. The expansion of therapy to these groups represents a critical advance, as interferon-based regimens were previously either poorly tolerated or contraindicated. In addition, the pediatric approval of DAAs has markedly broadened treatment access for younger populations. Oral, short-course, and well-tolerated regimens are now available for adolescents and children, closing a long-standing treatment gap and reinforcing the goal of HCV elimination across all age groups [80].

### 7.4. Remaining Challenges

Despite the remarkable advances achieved with DAAs, several barriers continue to hinder progress toward global HCV elimination. The cost of therapy remains a major obstacle, particularly in low- and middle-income countries where access to generic formulations is limited or unevenly distributed. Even where DAAs are available, health system constraints such as inadequate screening infrastructure, shortages of trained providers, and weak linkage-to-care pathways restrict their widespread implementation [81].

Another major challenge is reinfection among PWID, a population that bears a disproportionate burden of new infections. Reinfection risk highlights the need for integrated harm-reduction strategies, including needle–syringe exchange programs, opioid substitution therapy, and behavioral interventions. In addition, the lack of rapid, affordable point-of-care diagnostics delays case identification, particularly in resource-limited settings, where centralized laboratory testing may not be feasible [82].

Perhaps the most critical gap in long-term elimination strategies is the absence of a prophylactic vaccine. While DAAs can cure existing infection, they do not prevent reinfection. The extraordinary genetic diversity of HCV, coupled with its ability to evade humoral and cellular immunity, has complicated vaccine development. Without a preventive vaccine, elimination efforts will continue to rely on a combination of screening, treatment, and harm reduction, which may not be sufficient to achieve sustained eradication on a global scale [83].

## 8. Public Health and Policy Perspectives

The remarkable progress in HCV therapeutics has shifted the global conversation from disease management to the prospect of elimination. Yet treatment advances alone are insufficient to halt transmission or reduce the long-term public health burden. Effective elimination requires coordinated policy frameworks, equitable resource allocation, and sustainable integration of screening, treatment, and prevention strategies into existing healthcare systems [5,84].

A central challenge lies in the unequal distribution of diagnostic and therapeutic resources. High-income countries have achieved near-universal access to DAAs, while many low- and middle-income countries face barriers including high medication costs, limited laboratory infrastructure, and insufficient healthcare workforce capacity [85]. Even within well-resourced systems, marginalized groups such as PWID, incarcerated individuals, migrants, and the uninsured often remain underserved [86]. Addressing these disparities requires targeted outreach, simplified care pathways, and policy mechanisms that explicitly prioritize vulnerable populations.

Screening and linkage-to-care programs represent another major bottleneck. Although both the WHO and the U.S. Centers for Disease Control and Prevention (CDC) recommend universal one-time HCV testing in adults, implementation remains inconsistent [5,85]. In practice, many diagnoses occur late, when individuals present with advanced fibrosis or HCC. Streamlined diagnostic algorithms, such as reflex RNA testing and point-of-care technologies, have proven cost-effective and scalable, particularly in community-based and resource-limited settings. Embedding HCV testing into existing health infrastructures including antenatal care, HIV and tuberculosis programs, and primary care networks offers a pragmatic approach to maximizing diagnostic reach [5,85].

At the policy level, several national initiatives highlight the impact of political will. Egypt’s mass-screening and treatment campaign achieved millions of screenings and treatments within a few years, driving measurable declines in prevalence [84]. In high-income contexts, micro-elimination strategies targeting defined high-risk populations have also demonstrated success [86]. By setting achievable, population-specific goals, micro-elimination can serve as a pragmatic stepping stone toward broader national or regional elimination.

The success of these approaches, however, depends on sustained investment and political prioritization. Although the WHO declared viral hepatitis elimination a global target for 2030, funding commitments and national implementation plans remain uneven. Competing health priorities, constrained budgets, and fragmented governance structures often undermine momentum [82,83]. Stigma linked to HCV and its primary transmission routes further limits public engagement and discourages affected individuals from seeking testing and care [87].

Emerging digital health innovations offer additional tools to address gaps in the HCV care cascade. Telemedicine platforms, mobile health applications, and community-based self-testing strategies may reduce barriers in underserved areas and facilitate long-term monitoring of patients with advanced liver disease [84,85,86]. Their effectiveness, however, relies on adequate infrastructure, digital literacy, and supportive regulatory environments [88].

In summary, public health and policy perspectives indicate that HCV elimination is not solely a biomedical challenge but a multifaceted social and political endeavor. A successful strategy requires harmonizing therapeutic advances with robust preventive measures, equitable access, and systemic integration of screening and treatment. Without addressing the structural and policy-level barriers that perpetuate inequities, the transformative potential of DAAs will remain only partially realized.

## 9. Prevention and Future Directions

The advent of DAAs has transformed HCV management, yet prevention remains the most cost-effective and sustainable strategy to curb transmission and achieve global elimination. The persistence of new infections, together with reinfection in high-risk groups, foreground the need for integrated preventive measures alongside therapeutic advances [5,89].

### 9.1. Primary Prevention: Interrupting Transmission

Primary prevention aims to reduce new infections through targeted interventions. In high-income countries, transmission occurs predominantly among PWID. Evidence demonstrates that needle and syringe programs, opioid substitution therapy, and education on safe injection practices substantially reduce incidence, although these measures remain underutilized due to political, social, and financial barriers [90].

In low- and middle-income countries, healthcare-associated transmission continues to play a major role. Unsafe practices, including reuse of needles, inadequate sterilization of medical equipment, and unscreened blood transfusions, contribute significantly to ongoing spread [91]. Strengthening infection control, enforcing single-use injection devices, and ensuring access to safe blood supplies are essential strategies for prevention in these settings [91].

Mother-to-child transmission, while less common than with HBV or HIV, remains clinically relevant. At present, no standard prophylaxis exists, though research into the safety and efficacy of DAAs during pregnancy may open new avenues for perinatal prevention [92].

### 9.2. Secondary Prevention: Screening and Early Diagnosis

Because most HCV infections remain asymptomatic until advanced liver disease develops, early diagnosis is central to prevention. Both the WHO and the CDC recommend universal, one-time screening in adults, with repeat testing for high-risk populations including PWID, incarcerated individuals, and patients receiving chronic hemodialysis [93].

Simplified diagnostic tools such as dried blood spot assays, reflex RNA confirmation following antibody positivity, and point-of-care molecular platforms have shortened the time from diagnosis to treatment. Integration of HCV testing into HIV, tuberculosis, and antenatal care programs has proven highly effective in expanding reach, especially in resource-limited settings [94].

### 9.3. Reinfection and the Role of Harm Reduction

Even after achieving SVR, reinfection remains a significant challenge in high-risk groups such as PWID and men who have sex with men with high-risk sexual practices [89]. Mathematical modeling indicates that scaling up harm reduction services, including opioid substitution therapy and comprehensive addiction care, can reduce new infections by more than 50 percent [95].

Sustained behavioral interventions, peer-led education, and integration of substance-use treatment into HCV care models are essential to curb reinfection. Without parallel investment in harm reduction, the gains achieved by DAAs risk being eroded by cyclical transmission dynamics [95].

### 9.4. Vaccine Development

The development of an effective vaccine remains a critical unmet need. Extraordinary genetic heterogeneity, rapid mutation, and immune evasion have thwarted traditional vaccine approaches. However, novel platforms inspired by advances during the COVID-19 pandemic offer renewed promise. Strategies under investigation include messenger RNA vaccines, T cell-directed approaches targeting conserved epitopes, and passive immunization with broadly neutralizing antibodies. Early-phase trials have demonstrated immunogenicity, though durable and cross-genotypic protection has yet to be achieved. A partially effective vaccine, if combined with DAAs and harm reduction, could substantially reduce incidence and accelerate progress toward elimination [83].

### 9.5. Global Elimination Targets and Barriers

In 2016, the WHO set targets to eliminate HCV as a public health threat by 2030, defined as a 90 percent reduction in new infections and a 65 percent reduction in HCV-related mortality [5]. Yet most countries remain off track. Barriers include high drug costs in regions without affordable generics, weak diagnostic infrastructure, persistent healthcare inequities, and insufficient political prioritization [96].

Micro-elimination strategies, which focus on specific high-risk groups such as PWID, prisoners, or individuals with HIV, have shown measurable success and may provide scalable pathways toward broader elimination [96]. Egypt’s national mass screening and treatment campaign remains a landmark example of political commitment translating into population-level impact [5].

### 9.6. Future Perspectives

The future of HCV elimination depends on integrating biomedical innovation with social and policy interventions. Expanding access to generics, embedding HCV testing into routine health services, and deploying point-of-care RNA diagnostics will be crucial to simplify the care cascade [94]. Digital health platforms, mobile applications, and telemedicine offer new opportunities to expand linkage to care, particularly in underserved areas [96].

At the same time, reducing stigma and embedding community-led initiatives into elimination programs will be essential to maximize public engagement and improve long-term outcomes. Ultimately, a combined approach of universal screening, affordable treatment, robust harm reduction, and vaccine development represents the most realistic path to elimination. Without coordinated global investment and political will, however, the WHO 2030 targets risk remaining aspirational [96].

## 10. Conclusions

The landscape of HCV infection has been fundamentally transformed by the development of DAAs, which achieve cure rates exceeding 95 percent with short, well-tolerated regimens. Despite these therapeutic advances, HCV continues to represent a major global health burden, remaining a leading cause of cirrhosis, HCC, and liver transplantation. Millions of individuals remain undiagnosed, treatment uptake is uneven, and reinfection persists in high-risk populations, limiting the impact of current strategies.

## Figures and Tables

**Table 1:** Global prevalence of HCV infection by regions and major transmission drivers.

Region/Country	Estimated Prevalence	Primary Drivers of Transmission	Key Notes
Egypt	>10%	Historic PAT ^1^ campaigns	Highest national prevalence
Pakistan	5–7%	Unsafe injections	Large national burden
Sub-Saharan Africa	Variable (2–8%)	Medical transmission	Limited surveillance
U.S.	0.9–1.6%	Injection drug use	Increases among young adults
EU/EEA ^2^	6.2 per 100,000	Mixed (PWID + healthcare)	Regional variation
Australia	1%	PWID	Strong elimination programs

^1^ Parenteral Antischistosomal Therapy. ^2^ European Union/European Economic Area.

**Table 2:** Global distribution and virological characteristics of major HCV genotypes.

Genotype	Primary Geographic Distribution	Key Virological Features	Clinical/Therapeutic Considerations
1	North America, South America, Western Europe	Most common genotype worldwide; moderate genetic diversity	Historically low response to interferon; excellent SVR with DAAs
2	North America, West Africa	Less genetically diverse; stable evolutionary pattern	Generally favorable response to DAAs
3	South Asia; Europe (mainly among PWID)	Associated with steatosis; rapid quasispecies evolution	Higher fibrosis progression risk; slightly lower SVR in advanced liver disease
4	Middle East, North Africa, Sub-Saharan Africa	High intra-genotype variability, particularly in Egypt	Good DAA response; major contributor to regional disease burden
5	South Africa	Limited global distribution; low genetic diversity	Responds well to DAAs; clinical data relatively limited
6	Southeast Asia	Highly diverse; multiple subtypes	High SVR rates with pan-genotypic DAAs
7	Central Africa	Newly characterized genotype	Limited clinical data; early reports suggest responsiveness to modern DAAs

## Data Availability

No new data were created or analyzed in this study.

## Abbreviations

The following abbreviations are used in this manuscript:

HCV	hepatitis C virus
HCC	hepatocellular carcinoma
DAAs	direct-acting antivirals
SVR	sustained virologic response
PWID	people who inject drugs
WHO	World Health Organization
HBV	hepatitis B virus
HIV	human immunodeficiency virus
SR-B1	scavenger receptor class B type I
ER	endoplasmic reticulum
VLDL	very-low-density lipoprotein
MAVS	mitochondrial antiviral signaling protein
RIG-I	retinoic acid-inducible gene I
IRF3	interferon regulatory factor 3
MC	mixed cryoglobulinemia
CDC	Centers for Disease Control and Prevention
